# Safety and Immunogenicity of the Inactivated COVID-19 Vaccine Booster in People Living with HIV in China

**DOI:** 10.3390/vaccines11061019

**Published:** 2023-05-23

**Authors:** Yunyun Yi, Xiaoxu Han, Xinyu Cui, Peng Wang, Xin Wang, Hui Liu, Yuqi Wang, Na Zhu, Yanyan Li, Yingying Lin, Xin Li

**Affiliations:** 1 Center of Integrative Medicine, Beijing Ditan Hospital, Capital Medical University, Beijing 100015, China; 2 Beijing Key Laboratory for HIV/AIDS Research, Clinical and Research, Center for Infectious Diseases, Beijing Youan Hospital, Capital Medical University, Beijing 100069, China; 3 Center of Integrative Medicine, Peking University Ditan Teaching Hospital, Beijing 100015, China

**Keywords:** HIV, COVID-19, vaccines, immunogenicity, safety, third dose

## Abstract

Current knowledge regarding the long-term humoral response of people infected with human immunodeficiency virus to the third dose of inactivated coronavirus disease (COVID-19) vaccine is incomplete. As a result, concerns remain about the safety and efficacy of the vaccination. To improve our understanding of the safety and immunogenicity of the COVID-19 inactivated vaccine booster in people living with HIV (PLWH), a prospective study was conducted on participants who had not yet received a third dose of the COVID-19 inactivated vaccine, had no history of SARS-CoV-2 infection, and had received a second dose of the vaccine more than six months prior. The primary safety outcomes included the incidence of adverse reactions, changes in CD4^+^ T-cell count, viral load, blood routine examination, liver and kidney function examination, blood sugar, and blood lipid examination. The pseudovirus-neutralizing antibody responses to the D614G variant, Delta variant, and Omicron variants BA.5 and BF.7 were evaluated before vaccination, 14 days, 28 days, 3 months, and 6 months after vaccination to evaluate the immune response of PLWH to the injection of inactivated vaccine booster and the safety of the vaccine. In conclusion, COVID-19 vaccine booster shots were effective in PLWH, resulting in an increase in the number of CD4^+^ T-cells, neutralizing antibodies that lasted up to six months, and higher levels of neutralizing antibodies lasting approximately 3 months. However, the vaccine protection against the two variants of BA.5 and BF.7 was significantly lower than that of D614G and Delta.

## 1. Introduction

In the wake of the COVID-19 pandemic, research has indicated that HIV-infected patients often exhibit a poorer prognosis concerning COVID-19, with higher rates of severe disease and a higher likelihood of hospitalization as the stages of HIV disease progress [1]. Not only is the prognosis of COVID-19 poor, but SARS-CoV-2 is easy to spread among AIDS patients; compared to healthy people, AIDS patients suffer more from COVID-19, and their positive duration is longer. A study shows that adults with HIV over the age of 35 are more likely to spread SARS-CoV-2; thus, HIV infection may increase the spread of COVID-19 [2].

In addition to being highly transmissible, SARS-CoV-2 is also highly prone to mutation. A recent study identified 20 additional mutations in a variant SARS-CoV-2 detected in a 22-year-old female AIDS patient with uncontrolled advanced HIV infection who had been persistently infected with the beta variant of the virus for 9 months [3].

Immunization through vaccination has been widely recognized as the most effective and cost-efficient means of managing the COVID-19 pandemic [4,5,6,7,8]. Administration of inactivated vaccines has been shown to generate a robust immune response, resulting in the production of neutralizing antibodies that can provide protection against SARS-CoV-2 infection and alleviate symptoms in infected individuals [9,10]. Over time, the levels of antibodies within HIV patients have been observed to decrease at a faster rate, resulting in a corresponding decline in the efficacy of humoral immune protection [11]. Consequently, there is a global consensus to recommend a third dose of the COVID-19 vaccine. Amongst healthy individuals, those who received the third dose of the mRNA vaccine exhibited a lower incidence of SARS-CoV-2 infection during follow-up compared to those who did not receive the third dose [12]. In people living with HIV, it has been observed that the level of neutralizing antibodies produced shortly after the administration of the third dose of the COVID-19 vaccine is higher than that observed after the second dose [13]. Limited data are available on the long-term effectiveness and safety of the humoral immune response induced by the third dose of inactivated COVID-19 vaccine. Therefore, a study is required to investigate the efficacy and safety of vaccine boosters in people living with HIV.

Adaptive immunity is a critical component of the immune response against viral pathogens, with CD4^+^ T-cells playing a pivotal role in this process. During the response to SARS-CoV-2, virus-specific CD4^+^ T-cells can differentiate into various cell types that perform a range of auxiliary and effector functions. Among these functions are the actions of Tfh cells, which aid in the affinity maturation and production of antibodies by B cells. Th1 cells can directly suppress viral activity by secreting cytokines and recruiting innate immune cells, while CD4^+^ T-cells help in the proliferation and differentiation of CD8^+^ T-cells. In addition, CD4^+^-CTL can directly target virus-infected cells through class II antigen-restricted presentation, thus exerting cytotoxic activity [14].

Although antiretroviral therapy (ART) can suppress viral replication and partially restore immune function in people living with HIV, persistent CD4^+^ T-cell depletion is still a common occurrence. Our previous research indicated that individuals with CD4^+^ T-cell count ≤ 350 cells/μL had lower levels of SARS-CoV-2 IgG in response to inactivated vaccination, specifically against the D614G variant, compared to controls. This suggests that the efficacy of inactivated vaccination against COVID-19 may be reduced in PLWH with depleted CD4^+^ T-cell counts [15]. Numerous studies have shown that vaccine booster shots are capable of inducing a robust immune response. However, it is yet to be determined whether the aforementioned observation persists with the administration of a third shot in people living with HIV and whether it holds for different SARS-CoV-2 strains.

On 7 December 2022, following a re-adjustment of China’s public health control measures, SARS-CoV-2 infection became widely prevalent throughout the Chinese mainland. The predominant variants present in the region were BA.5 and BF.7, which had previously been recognized as the Omicron variant that had spread worldwide. These variants have been shown to exhibit an improved ability to evade immune responses, thus presenting a significant challenge to the effectiveness of current vaccines [16,17,18]. Recent research findings indicate that administering a booster dose has been shown to markedly mitigate neutralizing antibody responses against variants of concern (VOCs) [19,20]. Although breakthrough infections may occur despite a booster vaccination, it remains uncertain whether a fourth dose would be required to enhance immunological imprinting in such individuals.

In this study, we analyzed the safety and immunogenicity of inactivated vaccine booster injections in a prospective cohort study. It was expected to serve as a reference for guiding the timing of inactivated vaccine booster injections in HIV patients and whether booster injections in response to variant strains are necessary. The safety of the vaccine was assessed by adverse reactions and laboratory tests up to 28 days after booster vaccination, while the immunogenicity of the booster vaccine was assessed by changes in neutralizing antibody levels 6 months after vaccination.

## 2. Materials and Methods

### 2.1. Study Design and Recruitment of Cases

This study presents a prospective cohort of PLWH who were recruited from the outpatient clinic of the Department of Infection at Beijing Ditan Hospital between November 2021 and September 2022. This study was approved by the Ethics Committee of Beijing Ditan Hospital (No.: 2021-021-02). Participants met the following inclusion criteria: (1) PLWHs who had received the second dose of inactivated COVID-19 vaccine for more than six months without receiving the third dose; (2) confirmed HIV diagnosis by Western blot; and (3) signed informed consent. Exclusion criteria were: (1) age < 18 years; (2) history of SARS-CoV-2 infection; (3) malignant tumor or severe opportunistic infection; and (4) intellectual or language disorders. The third dose of vaccine administered to participants was the same as the second dose. This study was carried out in accordance with the Declaration of Helsinki.

### 2.2. Data Collection

Upon obtaining written informed consent from eligible participants, project staff facilitated the screening process to ensure adherence to study inclusion criteria. The participants were then directed to complete a privacy-protected questionnaire using a QR code link, administered both three days and one month after their third vaccination. Demographic information and any reported adverse reactions after vaccination were captured through this questionnaire, with the causal relationship of reported adverse events determined by an experienced physician. Venous blood was collected to assess antibody titers, CD4^+^ and CD8^+^ T-cell counts, HIV viral load, blood routine, liver and kidney function, blood lipid levels, and blood glucose levels. Follow-up appointments were scheduled on the 14th day, day 28, and month 6 after the third vaccination to collect additional venous blood samples for testing. Laboratory analysis of CD4^+^ T-cell counts and CD8^+^ T-cell counts, HIV viral load, blood routine, liver and kidney function, blood lipid levels, and blood glucose levels were performed in the Department of Laboratory Medicine of Beijing Ditan Hospital, with test results subsequently recorded in the hospital outpatient case system.

### 2.3. SARS-CoV-2 Antibody Measurement

In this study, we used a pseudovirus neutralization assay to assess the neutralizing antibody responses of all participants to D614G variant, Delta variant, and Omicron variants BA.5 and BF.7. Briefly, we used the SARS-CoV-2 pseudotyped virus kit (purchased from Zhongyanguobang Biological Technology Co., Ltd. in Beijing, China.) to assess the neutralizing antibody reactivity of all subjects to the above variants. After heat inactivation (56 °C, 30 min) of the serum samples, the serum samples were serially diluted with DMEM (6 times in a 3-fold gradient) and then incubated in duplicate with a certain amount (1300 TCID50/mL) of the pseudotyped virus in a 5% CO_2_ environment at 37 °C for 24 h. Both cell control (without virus and sample) and virus (without sample) controls were set up. After incubation, 50 µL of lysate per well was transferred to a white solid 96-well plate to lyse Vero cells for 10 min. A luciferase substrate (PerkinElmer, Waltham, MA, USA) was added to each well and incubated in the dark for two minutes. Relative luminescence units (RLU) per well were determined using a multifunctional enzyme marker (Thermo Fisher Technologies, Waltham, MA, USA). We calculated the median effective concentration (EC50) by using a nonlinear regression method, as described in a previous study [21,22], and determined the serum dilution that reduced RLU by 50% compared to the virus control well (virus and cells only). The titers of neutralizing antibodies were calculated as the reciprocal of the EC50 values, whereby a value of ≤10 was deemed non-neutralizing [22].

### 2.4. Statistical Analysis

The statistical analysis was conducted based on the basis of nature of the data. Continuous variables were presented as mean and range or the median and interquartile range (IQR). The normality assumption was tested for all continuous variables, and chi-squared variance analysis was performed. The paired sample *t*-test (for paired data) or the Wilcoxon test was used to compare continuous variables between two groups, while the Friedman test was used to compare repeated measurement data that did not meet the normal distribution assumption. Multiple comparison results were subjected to Bonferroni correction. After the logarithmic transformation of the antibody titer or concentration, the geometric mean titer (GMT) and its 95% confidence interval (CI) were calculated. SPSS version 25.0 software was employed for statistical analysis, and GraphPad Prism (version 9.5.0) was used for visualization. Statistical significance was considered when *p* < 0.05.

### 2.5. Vaccines

The COVID-19 vaccines used in this study were two inactivated vaccines (Vero cells), CoronaVac (Sinovac, Beijing, China) and Covilo (Sinopharm, Beijing, China). CoronaVac contains 3 µg in 0.5 mL of beta-propiolactone-inactivated SARS-CoV-2 from the CN02 strain grown in Vero, aluminum hydroxide as an adjuvant, and 0.5 mL injected [9,23]. Covilo contains 4 µg in 0.5 mL of beta-propiolactone-inactivated SARS-CoV-2 from the SARS-CoV-2019nCoV-CDC-Tan-HB02 strain grown in Vero, aluminum hydroxide as an adjuvant, and 0.5 mL injected [9,23].

## 3. Results

### 3.1. Baseline Characteristics

This study evaluated the clinical and demographic characteristics of people living with HIV (PLWH) who received COVID-19 vaccines, as well as the immunogenicity of the vaccines. The median age of the PLWH participants was 30 years (range 19–60), with a male predominance (97.56%). The mean weight and height of the PLWH were 68.1 kg (IQR 50–150 kg) and 173.27 cm (range 160–185 cm), respectively, and the median BMI was 21.97 (IQR 17.3–50). The median time before vaccination was 4 days (3–14 days), and the median duration of the four immunogenicity analyses after vaccination was 14 days (11–19 days), 28 days (24–32 days), 3 months (85–94 days), and 6 months (176–190 days). PLWH received combination antiretroviral therapy for more than six months, with non-nucleoside reverse-transcriptase inhibitors in combination with two nucleoside reverse-transcriptase inhibitors (NRTIs) being the most common treatment (48.20%). The Covilo vaccine was received by 58.5% of the participants, while the remaining 41.5% received the CoronaVac vaccine. In terms of comorbidities, PLWH with syphilis or condyloma acuminatum infection were 6 cases, respectively. The mean CD4^+^ T-cell count was 403 cells per μL (IQR 13–827 cells per μL), and 70.7% of HIV-infected patients were in a non-measurable viral load state. Some blood routine tests, liver and kidney function tests, and blood glucose tests were performed (Table 1). Statistical analysis was conducted using IBM SPSS 25.0 software, and the results showed that the difference was statistically significant with a *p*-value < 0.05.

### 3.2. Safety of Inactivated SARS-CoV-2 Booster Vaccines in PLWH

This study reports the overall incidence of adverse reactions within 7 days of vaccination in people living with HIV (PLWH) after receiving a booster vaccination. Of the 41 PLWH participants, 85.4% (35/41) experienced adverse events after vaccination. Injection site pain (53.7%) was the most frequently reported local event and generally resolved within 48 h. Injection site swelling (39.0%, 16/41), redness (39.0%, 16/41), and pruritus (14.6%, 6/41) were also commonly reported. Few systemic adverse events were reported, including drowsiness (14.6%, 6/41), muscle and joint pain (7.3%, 3/41), and nausea (0.8%, 2/41). All adverse events were self-reported by patients and were mild and self-limiting, with complete resolution within 7 days. No new adverse events occurred after 28 days of observation (Table 2).

In addition to clinical observations, laboratory data were collected before and after the booster vaccination to further assess the safety of the vaccine in PLWH. The results showed no significant changes in routine blood indices of leukocytes, red blood cells, and platelets within normal ranges after vaccination, as well as no changes in liver and kidney function indices, including alanine transaminase, glutamic oxalacetic transaminase, urea nitrogen, creatine, and uric acid within normal ranges. Additionally, there were no abnormal changes in triglycerides and glucose levels. These findings suggest that the booster vaccine is safe and does not cause significant adverse effects on laboratory parameters in PLWH (Table 3).

### 3.3. Changes in CD4^+^ T-Cell Counts

**Figure 1:**
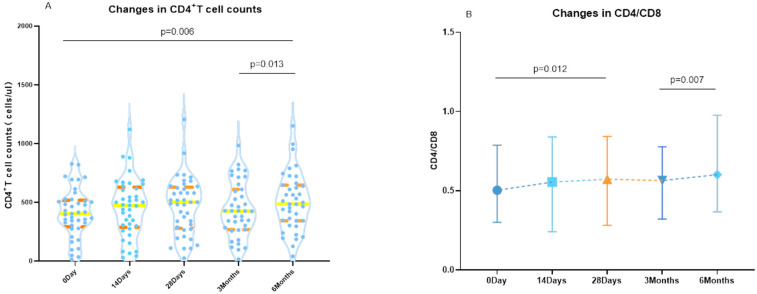
Changes in CD4^+^ T-cell counts and CD4^+^ T-cells/CD8^+^ T-cells over time in AIDS patients after vaccination. (**A**) The orange line represents the quartiles, the yellow line represents the median, and the scatter represents the CD4^+^ T-cell counts measured in each patient. (**B**) The changes in CD4^+^ T-cells/CD8^+^ T-cells over time in AIDS patients after vaccination. The points connected by dashes represent the median, and each horizontal coordinate corresponds to the highest and lowest points of the graph representing the quartiles.

Repeated measures ANOVA was conducted to analyze CD4 counts at various time points (i.e., before vaccination, 14 days, 28 days, 3 months, and 6 months after vaccination) in the study population. The Greenhouse–Geisser estimate of deviation from sphericity was calculated as ε = 0.914, which was statistically significant (*p* = 0.002). Subsequent multiple comparisons revealed that CD4^+^ T-cell counts were significantly higher than baseline levels at both 14 and 90 days post-vaccination (*p* = 0.003 and *p* = 0.016, respectively) (Figure 1A). Conversely, the Friedman test analysis showed no significant change in CD8^+^ T-cell counts at different time points (i.e., before vaccination, 14 days, 28 days, 3 months, and 6 months after vaccination), with a *p*-value of 0.156.

A Friedman test analysis was conducted on CD4^+^ T-cell and CD8^+^ T-cell counts at 14 days, 28 days, 3 months, and 6 months post-vaccination, revealing a statistically significant difference (*p* < 0.001). Subsequent multiple comparisons revealed that the CD4^+^/CD8^+^ T-cell count was higher before vaccination compared to 28 days after vaccination (*p* = 0.012) and was also higher at 6 months after vaccination compared to 3 months (*p* = 0.007) (Figure 1B).

### 3.4. Neutralizing Activity of the Vaccine

**Figure 2:**
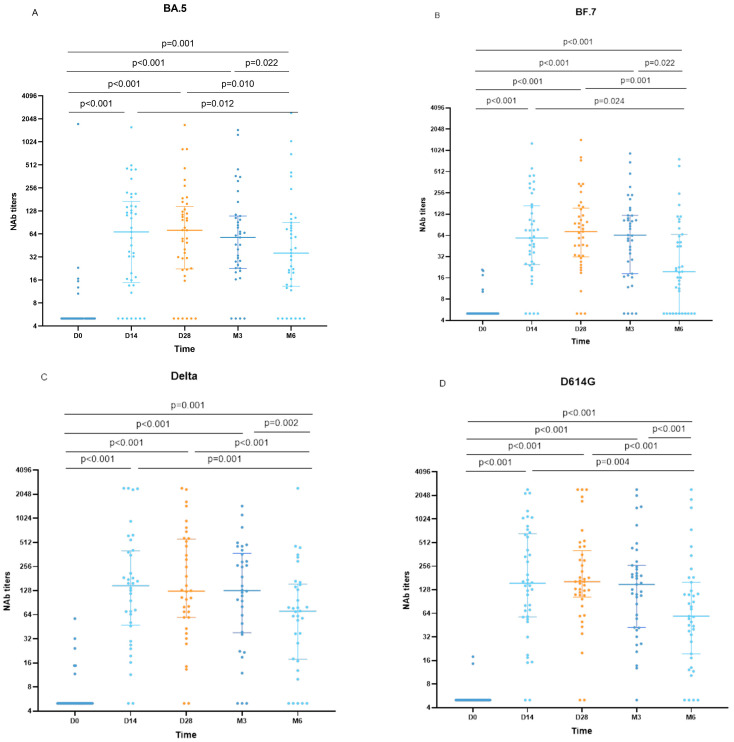

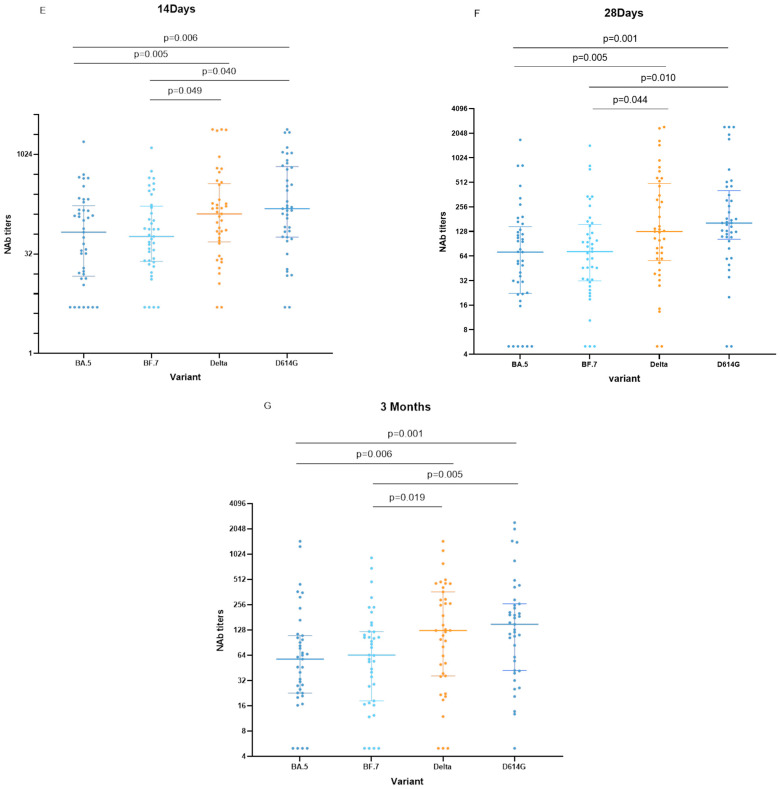
Neutralization activity of SARS-CoV-2 spike variants by plasma. D0, before booster vaccination; D14, 14 days after booster vaccination; D28, 28 days after booster vaccination; M3, 3 months after booster vaccination; M6, 6 months after booster vaccination. The longer horizontal line represents the median neutralizing antibody titers and the horizontal lines at the ends represent the quartiles of neutralizing antibodies titers. Dots indicate the neutralizing antibody titer for each participant, and dots outside the shorte horizontal line indicate dots beyond 1.5 times the IQR. (**A**) The neutralizing antibody levels of PLWH against BA.5 strain before the third vaccination and 14 days, 28 days, 3 months, and 6 months after the vaccination. (**B**) The levels of neutralizing antibodies detected in PLWH against BF.7 before and 14 days, 28 days, 3 months, and 6 months after the third vaccination. (**C**) The levels of neutralizing antibodies detected in PLWH against Delta strain before and 14 days, 28 days, 3 months, and 6 months after the third vaccination. (**D**) The levels of neutralizing antibodies detected in PLWH against the D614G strain before the third vaccination and 14 days, 28 days, 3 months, and 6 months after the vaccination. (**E**) The level of neutralizing antibodies against BA.5, BF.7, Delta, and D614G about 14 days after PLWH booster vaccination. (**F**) The neutralizing antibody levels against BA.5, BF.7, Delta, and D614G about 28 days after PLWH booster vaccination. (**G**) The neutralizing antibody levels against BA.5, BF.7, Delta, and D614G about 3 months after PLWH booster vaccination.

Four pseudoviruses were employed to bind with antibodies in the serum of AIDS patients at five-time points before and following vaccination. Our study revealed that 14.63% (6/41) of HIV patients possessed residual neutralizing antibodies against the BA.5 variant before the booster vaccination, while 82.92% (34/41), 85.36% (35/41), 89.74% (35/39), and 82.05% (32/39) of AIDS patients generated higher levels of neutralizing antibodies which could be detected at 14 days, 28 days, 3 months, and 6 months after vaccination, respectively. The pairwise comparison of the data with repeated measurements of non-normal distribution and Friedman’s test analysis demonstrated a significant difference between the neutralizing antibodies detected at 14 days, 28 days, 3 months, and 6 months after vaccination (*p* = 0.001, *p* < 0.001, *p* < 0.001, *p* < 0.001, respectively), and a considerable decrease in antibody titers at 6 months post-vaccination, with statistically significant differences from 14 days, 28 days, and 3 months post-vaccination (*p* = 0.001, *p* < 0.001, *p* = 0.002, respectively). However, antibody titers at 14 days, 28 days, and 3 months post-vaccination were statistically significant (*p* = 0.001, *p* < 0.001, *p* = 0.002, respectively). There were no significant differences in antibody titers detected after 14 days, 28 days, and 3 months after vaccination (*p* = 0.858, *p* = 0.252, *p* = 0.334) (Figure 2A).

This study investigated the neutralizing ability of antibodies in PLWH against the BF.7 variant at different time points before and after booster vaccination. Before the booster vaccination, 12.20% of HIV patients had residual neutralizing antibodies against the BF.7 strain, while the majority of patients generated higher levels of neutralizing antibodies, which could be detected after vaccination, with percentages of 90.24%, 92.68%, 89.74%, and 71.79% at 14 days, 28 days, 3 months, and 6 months post-vaccination, respectively. To analyze the data from repeated measurements with non-normal distribution, we utilized Friedman’s test and performed pairwise comparisons. Our results demonstrated that the neutralizing antibodies detected at each time point were significantly different and statistically significant (*p* < 0.001 for all). Furthermore, we observed a statistically significant decrease in antibody titers at 6 months post-vaccination compared to 14 days, 28 days, and 3 months post-vaccination (*p* = 0.024, *p* = 0.022, *p* = 0.001, respectively), while there was no significant difference in antibody titers between 14 days, 28 days, and 3 months post-vaccination (*p* = 0.971, *p* = 0.267, *p* = 0.283) (Figure 2B).

We assessed the neutralization ability of antibodies in PLWH against the Delta variant and observed that before booster vaccination, only a small proportion of HIV patients (14.63%; 6/41) had residual neutralizing antibodies against the Delta strain, whereas, after vaccination, a majority of patients (95.12%, 39/41) generated higher levels of neutralizing antibodies which could be detected at 14 days, 28 days, 3 months, and 6 months post-vaccination. The non-normally distributed data of repeated measurements were subjected to pairwise comparisons and analyzed by the Friedman test, which revealed statistically significant differences in neutralizing antibodies detected at pre- and post-vaccination time points (*p* = 0.001, *p* < 0.001, *p* < 0.001, *p* < 0.001, respectively). Further analysis showed that antibody titers significantly declined at 6 months after vaccination and were significantly different from those observed at 14 days, 28 days, and 3 months after vaccination (*p* = 0.001, *p* < 0.001, *p* = 0.002, respectively), whereas no significant differences were observed in antibody titers detected at 14 days, 28 days, and 3 months after vaccination (*p* = 0.858, *p* = 0.252, *p* = 0.334, respectively) (Figure 2C).

Regarding the capacity of antibodies in people living with HIV (PLWH) to neutralize the D614G variant, our study revealed that a small percentage (0.05%; 2/41) of HIV patients had residual neutralizing antibodies against the D614G strain before the booster vaccination, whereas the majority of patients (≥89.74%) generated higher levels of neutralizing antibodies which could be detected at various time points after vaccination. Analysis of the data using the Friedman test for pairwise comparison of repeated measurements with non-normal distribution showed no significant difference in the antibody titers detected at 14 days, 28 days, and 3 months after vaccination (*p* = 0.431, *p* = 0.133, *p* = 0.474, respectively). Nonetheless, neutralizing antibodies were detected and persisted at relatively high levels for up to 6 months after vaccination in most of the PLWH studied (Figure 2D).

This study aimed to examine the levels of neutralizing antibodies in AIDS patients following vaccination against different strains at 14 days post-vaccination. Our results revealed significantly lower levels of neutralizing antibodies detected against the BA.5 strain compared to the Delta and D614G strains (*p* = 0.006, *p* = 0.005, respectively). Furthermore, we observed lower levels of neutralizing antibodies detected against the BF.7 strain compared to those detected against the Delta and D614G strains (*p* = 0.040, *p* = 0.049, respectively) (Figure 2E).

Upon evaluating the levels of neutralizing antibodies produced by HIV/AIDS patients about 28 days after vaccination against different strains, we observed that the levels of antibodies detected against the BA.5 strain were significantly lower than those detected against the Delta and D614G strains (*p* = 0.001, *p* = 0.005, respectively). Additionally, we found that the levels of antibodies detected against the BF.7 strain were lower than those detected against the Delta and D614G strains (*p* = 0.010, *p* = 0.044, respectively) (Figure 2F).

In this study, we assessed the levels of neutralizing antibodies detected in AIDS patients approximately 3 months after vaccination against various strains. Our results indicated that the levels of antibodies detected against the BA.5 strain were significantly lower than those detected against the Delta and D614G strains (*p* = 0.006, *p* = 0.001, respectively). Furthermore, our data demonstrated that the levels of antibodies detected against the BF.7 strain were lower than those detected against the Delta and D614G strains (*p* = 0.019, *p* = 0.005, respectively) (Figure 2G).

**Figure 3:**
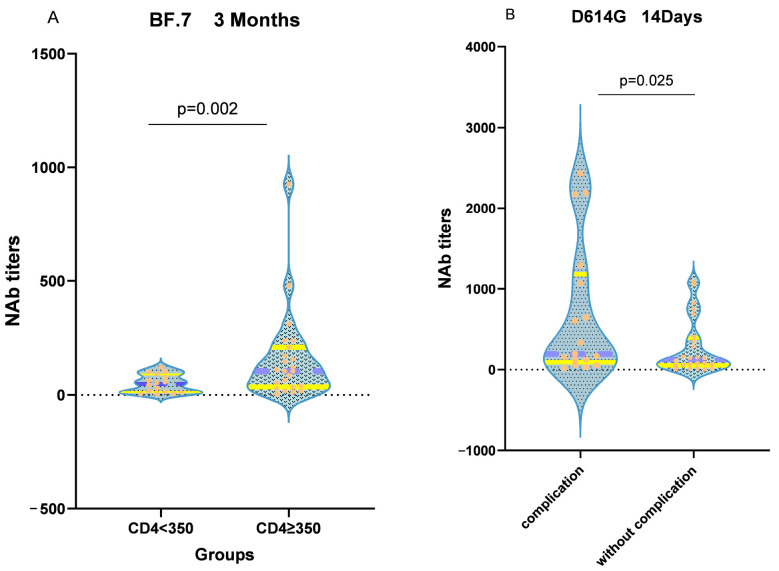
(**A**) CD4^+^ T-cell counts according to PLWH, divided into two groups at 350, with the yellow line representing the quartiles and the purple line representing the median. The black dashed line indicates a neutralizing antibody titer of 0. The orange dots represent the neutralizing antibody titers of the participants (**B**) Patients were divided into two groups according to whether they had AIDS complications or not, with the orange line representing the quartiles and the yellow line representing the median. The black dashed line indicates a neutralizing antibody titer of 0. The orange dots represent the neutralizing antibody titers of the participants.

In AIDS patients, CD4^+^ T-cells are severely impaired, but they are crucial for vaccine-induced humoral immunity. To evaluate the impact of CD4^+^ T-cell levels on neutralizing antibody responses, patients were divided into two groups based on their CD4^+^ T-cell counts: CD4^+^ T < 350 and CD4^+^ T ≥ 350. A Wilcoxon rank sum test was conducted, revealing that 3 months after vaccination, AIDS patients with CD4^+^ T ≥ 350 had higher levels of neutralizing antibodies against the BF.7 strain than those with CD4^+^ T < 350 (*p* = 0.02) (Figure 3A). Furthermore, patients with complications exhibited lower levels of neutralizing antibodies against the D614G strain at around 14 days after vaccination than those without complications (*p* = 0.025) (Figure 3B).

## 4. Discussion and Conclusions

The COVID-19 pandemic has continued for over three years since its initial outbreak in 2019. A recent study comparing the immune response of healthy individuals with those of individuals with solid organ transplants, rheumatic diseases, cancer, and HIV infection revealed a low rate of neutralizing antibody positivity following vaccination with the inactivated SARS-CoV-2 vaccine. Consequently, a third vaccine dose has been recommended to boost the immune response in these vulnerable populations [24]. The ongoing mutations of the SARS-CoV-2 virus pose a challenge to the development of effective vaccines. The newly emerged Omicron variant exhibits a robust immune escape capability, leading to a reduction in vaccine efficacy and antibody drug resistance [25,26,27]. Our findings are consistent with the work of Alexandra Tauzin [26] and demonstrate that the inactivated vaccine booster has a diminished capacity to neutralize BA.5 and BF.7 compared to the D614G and Delta strains in individuals living with HIV at the third vaccination. This indicates that the current vaccine, when used as a booster, elicits a range of antibodies that are more likely to be specific to the original SARS-CoV-2 strain. These results underscore the need to develop a new version of the vaccine that targets the Omicron variant, which may offer superior protection.

The compromised immune system of AIDS patients leads to the impairment of CD4^+^ T-cells, which in turn negatively affects their humoral immune response. Furthermore, because vaccines may not elicit a protective immune response in AIDS patients and may cause adverse reactions due to their weakened antigenicity, several studies have indicated that AIDS patients are more hesitant to receive vaccines compared to healthy individuals [28]. Prior research has demonstrated the safety of vaccines with minimal adverse effects in healthy individuals. However, evidence on the safety of vaccines for those with AIDS is limited, primarily comprising symptomatic reports with insufficient laboratory examination. In this study, we assessed the safety of the inactivated vaccine among AIDS patients by dynamically monitoring specific laboratory indicators, such as blood routine, liver and kidney function, blood glucose, and lipid levels, along with symptom-related questionnaires. Our findings suggest that the inactivated vaccine is safe for people living with HIV, as we did not observe any significant safety concerns one month after the booster vaccination. This is consistent with the findings of Kang et al. [29] for the first and second doses of inactivated vaccines. However, the sample size was limited, which warrants further investigation with larger sample sizes.

Through dynamic observation of CD4^+^ T-cell responses in PLWH, we found a significant increase in CD4^+^ T-cell counts at 6 months post-booster vaccination, compared to pre-vaccination and at 3 months after vaccination. This increase highlights the important role played by CD4^+^ T-cells in inactivated vaccine-induced adaptive immunity. It has been shown in animal models that the response of CD4^+^ T-cells to SARS-CoV-2 is more pronounced than that of CD8^+^ T-cells [30]. B-cell-depleted individuals were able to effectively clear SARS-CoV-2 exhibiting a strong clonal amplification and highly selective class ii specific CD4^+^ T-cell response [31]. The observed increase in CD4^+^ T-cell counts at 6 months post-vaccination may be attributed to a boost in memory CD4^+^ T-cell counts resulting from the development of memory immunity elicited by the booster vaccination. These findings are in line with the study conducted by Zuo et al. [32]. Concurrently, the potential impact of antiviral therapy on the CD4^+^ T-cell count rise cannot be disregarded.

In our study, a longer duration of neutralizing antibodies with higher titers was observed after booster inoculation compared to the second vaccine. This is similar to the results of Luczkowiak et al. [33] in the mRNA vaccine study. The peak time of the neutralizing antibodies was approximately 40 days, with a rapid decline in 40–60 days after the second vaccine, while a higher titer of neutralizing antibodies was found to last for approximately 90 days in PLWH after booster inoculation, with a sustained high neutralizing antibody response level lasting for six months. To compare the antibody level before and after the third shot, we assessed patients who received the third shot around six months after the second shot and found a statistically significant difference in the antibody level. Our findings are consistent with the research results of Lapointe et al. [13], which indicated that booster shots improved the intensity and prolonged the duration of immunization. Although we did not conduct a follow-up of the third dose of inactivated vaccine in healthy individuals, HIV patients in a Hong Kong study [34] compared with healthy individuals 14 days after the third dose of inactivated vaccine produced lower neutralizing antibodies than healthy individuals, and the same phenomenon was seen in the mRNA vaccine [35] and in previous studies [15,36,37] of the second dose. This may be related to impaired antigen-specific B and T-cell responses in PLWH [38].

Our study evaluated the effect of low CD4^+^ T-cell count on the immune response to booster shots, although, in studies of a second dose of inactivated vaccine, Wu et al. and Netto et al. found that the immune response to inactivated COVID-19 vaccination among [39,40]. However, we found this effect to be negligible in the inactivated vaccine booster dose. Although we did observe a modest effect on early humoral immunity to the Omicron variant BF.7. This could be attributed to the antibody profile elicited by the booster shots, which appears to be more focused on the original strain [41]. In a study conducted by Bessen et al. [35], it was observed that low CD4^+^ T-cell counts did not impede the cellular immune response in people living with HIV (PLWH). Even though the CD4^+^ T-cell counts were low, the SARS-CoV-2 vaccination led to a robust cellular immune response in PLWH [36,42]. The high-intensity humoral immune response observed in PLWH after booster vaccination could be attributed to the strong memory immunity generated even with low CD4^+^ T-cell counts. However, the small sample size used in this study calls for caution in the interpretation of the findings. Although the difference in the immune response between PLWH with and without complications was not significant, except for the early humoral immunity against the D614G variant at 14 days post-vaccination, the variability in the rate of early humoral immunity production among individuals may account for this result. Moreover, no significant difference was observed in the peak effect of humoral immunity.

This study is limited by several factors. Firstly, the inability to track patients who received the second vaccination before our study. The patients investigated in this manuscript are not the same group of patients as the previously published patients of the second injection [15], which is caused by two major reasons: firstly, before we recruited the subjects of the third injection study, there were already some patients of the second injection who had received the third injection in advance, and we could not obtain their baseline information before the injection; and secondly, because the third injection study required five follow-up visits, many patients who had received the second injection felt that they could not persist in the study due to transportation and scheduling reasons. Secondly, we did not perform a direct comparison of the immune response between the second and third vaccinations. Another limitation is the small sample size, as all participants were recruited from a single hospital, which may introduce bias. Furthermore, the recruitment of healthy individuals as controls was challenging due to the frequent follow-up requirements. Poor adherence to follow-up in healthy populations under conditions of epidemic containment in China. Additionally, we were unable to compare antibody production between healthy individuals and PLWH. Notably, our study did not investigate the latest subtypes of the globally prevalent Omicron strains, such as BQ.1 and XBB. Finally, our use of pseudovirus neutralization assays may not be as convincing as real virus neutralization experiments.

In conclusion, our study demonstrated that booster shots of COVID-19 vaccines were efficacious in individuals living with HIV (PLWH), as evidenced by the significant increase in CD4^+^ T-cell counts and sustained antibody levels for up to six months. Moreover, higher levels of neutralizing antibodies were observed for approximately three months following booster shots. Nonetheless, the efficacy of these booster shots against the BA.5 and BF.7 variants was significantly lower than that against the D614G and Delta variants.

## Figures and Tables

**Table 1:** Characteristics of participants.

Characteristics	PLWH (*n* = 41)
Age (years), median (range)	30 (19–60)
Gender	
Male, *n* (%)	40 (97.56)
Female, *n* (%)	1 (2.44)
Weight (kg), median (IQR)	66 (57–75)
Height (cm), mean (range)	173.27 (160–185)
BMI, median (IQR)	21.97 (17.30–50.70)
Days before 3rd dose Vaccination, median (range)	
4 (3–14), *n* (%)	41 (100)
Days after 3rd dose Vaccination, median (range)	
14 days (11–19 days), *n* (%)	41 (100)
28 days (24–32 days), *n* (%)	41 (100)
3 months (85–94 days), *n* (%)	39 (95.12)
6 months (176–190 days), *n* (%)	39 (95.12)
ART use, *n* (%)	
2NRTIs + NNRTIs	27 (48.20)
2NRTIs + INSTIs	11 (19.70)
2NRTIs + PIs	3 (5.40)
COVID-19 Vaccine, *n* (%)	
COVILO	24 (58.5)
CoronaVac	17 (41.5)
Time of HIV diagnosis, median (range), years	2 (0.5–9)
HIV viral load before vaccination, median (IQR), copies/mL	20 (20–29,509)
CD4_+_ T-cell counts before vaccination, median (IQR), cells/uL	403 (13–827)
CD8_+_ T-cell counts before vaccination, median (IQR), cells/uL	732 (432–2189)
CD4/CD8 ratio before vaccination, median (IQR)	0.50 (0.30–0.79)
Comorbidities, *n* (%)	
Herpes zoster	1 (2.4)
Herpes simplex	2 (4.8)
Sphagnum	1 (2.4)
Syphilis	6 (14.6)
Condyloma acuminatum	6 (14.6)
Tuberculosis	1 (2.4)
Pneumocystis carinii pneumonia	1 (2.4)
Thrush	1 (2.4)
Eczema	1 (2.4)
Diabetes	1 (2.4)
Hyperlipidemia	5 (12.2)
Chronic hepatitis	1 (2.4)
Hypertension	1 (2.4)
Laboratory examination	
White blood cell count (10^9^/L) median (IQR)	5.70 (4.60–6.86)
Red blood cell count (10^9^/L) median (IQR)	4.87 (4.50–5.20)
Platelet count (10^9^/L) median (IQR)	254 (213–274)
Aspartate aminotransferase (U/L) median (IQR)	25.40 (18.60–30.15)
Alanine aminotransferase (U/L) median (IQR)	28.60 (13.00–159.00)
Blood urea nitrogen (mmol/L) mean (range)	4.48 (2.92–6.93)
Serum creatinine (μmol/L) mean (range)	73.90 (44.40–105.70)
Triglyceride (mmol/L) median (IQR)	1.46 (0.87–2.87)
Uric acid (μmol/L) mean (range)	367.98 (205.00–603.00)
Blood glucose (mmol/L) median (IQR)	5.55 (5.27–5.78)

Abbreviations: NRTIs—nucleoside reverse-transcriptase inhibitors; NNRTIs—non-nucleoside reverse-transcriptase inhibitors; INSTIs—integrase inhibitors; PIs—protease inhibitors; PLWH—people living with HIV.

**Table 2:** Adverse events of COVID-19 vaccination in participants.

Variable	PLWH (*n* = 41)
Overall adverse events within 7 days	35 (85.4%)
Overall adverse events within 28 days	35 (85.4%)
**Local adverse events**	
Pain	22 (53.7%)
Redness	16 (39.0%)
Itch	6 (14.6%)
Induration	/
Swelling	16 (39%)
**Systemic adverse events**	
Lethargy	6 (14.6%)
Fever	/
Headache	/
Muscle and joint pain	3 (7.3%)
Nausea	2 (4.9%)
Thrombotic events or bleeding	/

**Table 3:** Changes in laboratory examination.

Variable	Days before 3rd Dose of Vaccination	28 Days after 3rd Dose of Vaccination	*p*-Value
White blood cell count (10^9^/L) median (IQR)	5.70 (4.60–6.86)	5.45 (4.8–6.15	0.323
Red blood cell count (10^9^/L) median (IQR)	4.87 (4.50–5.20)	4.83 (4.56–5.17)	0.835
Platelet count (10^9^/L) median (IQR)	254 (213–274)	243 (220.5–280)	0.766
Aspartate aminotransferase (U/L) median (IQR)	25.40 (18.60–30.15)	21.10 (16.95–28.8)	0.389
Alanine aminotransferase (U/L) median (IQR)	28.60 (19.50–43.75)	27.60 (16.7–38.20)	0.424
Triglyceride (mmol/L) median (IQR)	1.46 (0.87–2.87)	1.30 (0.98–2.67)	0.973
Blood urea nitrogen (mmol/L) mean (range)	4.48 (2.92–6.93)	4.74 (2.70–8.25)	0.163
Serum creatinine (μmol/L) mean (range)	73.90 (44.40–105.70)	76.86 (53.30–101.70)	0.073
Uric acid (μmol/L) mean (range)	367.98 (205.00–603.00)	387.56 (222.00–620.00)	0.265
Blood glucose (mmol/L) median (IQR)	5.55 (5.265–5.78)	5.38 (5.06–5.69)	0.492

## Data Availability

The corresponding author can provide access to the datasets generated and/or analyzed during this study upon reasonable request.

## Abbreviations

PLWH—people living with HIV; COVID-19—coronavirus disease 2019; SARS-CoV-2—severe acute respiratory syndrome coronavirus 2; ART—antiretroviral therapy; IQR—interquartile range; GMTs—geometric mean titers; CIs—confidence intervals.; VOC—variants of concern. TCID_50_—the 50% tissue culture infectious dose.
